# Assessing suicidality in adult ADHD patients: prevalence and related factors: Suicidality in adult ADHD patients

**DOI:** 10.1186/s12991-024-00528-8

**Published:** 2024-11-01

**Authors:** Gabriele Di Salvo, Camilla Perotti, Lorenzo Filippo, Camilla Garrone, Gianluca Rosso, Giuseppe Maina

**Affiliations:** 1 Department of Neurosciences “Rita Levi Montalcini”, University of Turin, Turin, Italy; 2 Psychiatric Unit, San Luigi Gonzaga University Hospital, Orbassano, Turin, Italy

**Keywords:** Attention-deficit hyperactivity disorder, Suicidal ideation, Suicidal behavior, Suicide attempts, Risk factors

## Abstract

**Background:**

The association between Attention-deficit hyperactivity disorder (ADHD) and suicidality has been subject of growing interest for research in the latest years. Suicidality was generally assessed categorically and without the use of validated instruments, leading to heterogeneous or even conflicting evidence. The prevalence of both suicidal ideation and attempts varies considerably, and the associated risk factors remain unclear. Our study investigated suicidality in ADHD using a dimensional approach and a validated and internationally recognized instrument. Our primary aim was to evaluate the prevalence of suicidal ideation (SI), severe suicidal ideation (SSI), suicidal behavior (SB) and non suicidal self-injury behavior (NSSIB) in a sample of adult patients with ADHD. The second objective was to identify sociodemographic and clinical features associated with increased risk of suicidality in these patients.

**Methods:**

The sample included 74 adult patients with clinical diagnosis of ADHD. Suicidality was assessed by administering the Columbia-Suicide Severity Rating Scale. Logistic regressions were used to examine predictors of SI, SSI, SB and NSSIB.

**Results:**

The lifetime prevalence of SI and SSI were 59.5% and 16.2%, respectively. The 9.5% of patients showed lifetime SB, while NSSIB was found in 10.8% of the subjects. Lifetime SI was associated with severity of inattentive symptoms during adulthood, low self-esteem and impairment in social functioning. Lifetime SSI appeared related to severity of inattentive symptoms during childhood, attentional impulsiveness and number of hospitalizations, while physical activity appeared to be protective. The prevalence of lifetime SB and NSSIB did not appear significantly related to any socio-demographic or clinical feature.

**Conclusions:**

Adults with ADHD should be considered at risk of suicide and it is important to determine which patients are at higher risk, in order to guide preventive interventions. The association between ADHD and suicidal ideation did not appear to be influenced by psychiatric comorbidities, but rather by inattention itself, which represents the core symptom of ADHD.

## Introduction

Attention-deficit hyperactivity disorder (ADHD) is a neurodevelopmental condition that begins in childhood and frequently persists into adulthood [1]. *ADHD has an estimated prevalence of 4–7% in childhood* [2] *and of 2.5% in adulthood* [3]. ADHD symptoms encompass inattention and hyperactivity, as well as impulsivity and difficulties in emotional regulation [4]. Furthermore, ADHD often co-occurs with other psychiatric disorders such as mood disorders, substance use disorder and personality disorders [5].

Consequently, the link between ADHD and suicidality has garnered significant research interest in recent years. *When discussing suicidality*,* we refer to a complex phenomenon that includes suicidal ideation (SI)*,* severe suicidal ideation (SSI)*,* suicidal behavior (SB) and non suicidal self-injury behavior (NSSIB). According to a recent meta-analysis*,* lifetime SI and SB prevalences in ADHD adult patients are 40% and 18.9%*,* respectively* [6].

Nevertheless, several limitations have led to heterogeneous or even conflicting findings in the literature. The first methodological limitation lies in the conceptualization and categorical assessment of suicidality, as suicide is a complex and dynamic phenomenon that goes from SI to SB. Thus, specific evaluation tools, like the Columbia-Suicide Severity Rating Scale (C-SSRS), are needed as they enable a dimensional analysis of suicidality 7 [4]. Nonetheless, most research on suicidality in ADHD has employed open-ended questions or questionnaires that were neither specifically designed nor validated for this purpose, or solely relied on diagnostic codes to assess suicidality. We identified only three studies involving adult patients where the C-SSRS or other validated instruments were used [8–10].

Furthermore, while several studies documented the incidence of suicide attempts in ADHD, there is a lack of evidence on other dimensions of suicidality, such as SI, and on clinical or sociodemographic features which may moderate the risk for suicide in ADHD.

Considering the heterogeneous clinical presentation (which, especially in adults, goes far beyond inattention and hyperactivity), the evolving nature of the symptoms, and the high prevalences of psychiatric comorbidities that could complicate or delay the detection of the disease, ADHD diagnosis represents a challenge for clinicians [11, 12]. Furthermore, it is known that certain dimensions of psychiatric disorders (such as specific affective temperaments, coping strategies and defense mechanisms) represent risk factors for suicidality, independently from the disease itself [13, 14]. For these reasons, it should be important to assess ADHD patients following a dimensional approach, as many of dimensions of ADHD could affect the risk of complications [15, 16]. However, most of the studies available not only do not take into account these dimensions, but also do not rely on clinical diagnosis. Indeed, certain studies did not employ clinical or diagnostic interviews, relying exclusively on non-specific screening tools, diagnostic codes, or stimulant prescriptions to identify patients with ADHD [9, 17, 18]. Furthermore, a substantial portion of the research focused solely on male or underage patients, as well as specific populations (such as prison inmates, substance abusers, and individuals with learning disabilities) [19–21].

These methodological limitations and differences in studies design and samples make the results difficult to interpret and compare, besides generating a considerable variability between the results themselves. In literature lifetime SB in adult ADHD patients rates range from 9.1% [21] to 51.5% [22], while lifetime SI rates range from 15.8% [23] to 66.3% [20].

Some researchers have examined risk factors for suicidality in ADHD, with gender being one of the most widely explored factors. The majority of studies have indicated a higher risk of both suicidal ideation (SI) and suicidal behavior (SB), but not of completed suicide, in adult females with ADHD [17, 24–26]. On the other hand, no significant differences were found among underage patients [28, 29].

Few data regarding the potential impact on suicidality of other important dimensions, such as age at diagnosis, symptom severity, ADHD subtype, related symptoms and impulsiveness, is available. Only recently, a meta-analysis underlined ADHD symptoms severity and persistence, family history of ADHD, parental influences and social functioning as risk factors for suicidality in adult patients with ADHD [24].

Concerning the potential influence of psychiatric comorbidities on the relationship between ADHD and suicidality, Septier and colleagues conducted a meta-analysis that highlighted an association largely independent of such variables (including psychiatric comorbidities) [6]. Other studies have suggested a generally significant association even when adjusting for socio-demographic and clinical factors [17, 19, 25]. However, some studies did not replicate these findings [8, 9, 20, 30]. Therefore, the prevalence of suicidality in ADHD and factors related remain unclear, as it remains controversial whether this association is direct or mediated by psychiatric comorbidities.

Our study investigated suicidality in ADHD using a dimensional approach and a validated and internationally recognized instrument. Our primary aim was to evaluate the prevalence of SI, SSI, SB, and NSSIB in a sample of adult patients with ADHD. The second objective was to identify sociodemographic and clinical features associated with increased risk of suicidality in these patients.

To achieve these objectives, we recruited a sample of adult patients with ADHD assessed with a dimensional approach, aiming to overcome, or at least mitigate, the aforementioned methodological limitations. Regarding the sample size, it is important to note that ADHD is a highly specialized topic, and its diagnosis and treatment in Italy can only be carried out by licensed psychiatrists. Therefore, referring patients with ADHD is not as straightforward as with other psychiatric disorders, and this must be taken into account when considering the sample sizes of studies (which is usually smaller than for other disorders).

A better understanding of not only the prevalence of suicidality in ADHD, but also whether it is influenced by other psychiatric disorders or certain clinical dimensions of ADHD, could help identify patients at risk of suicide and, consequently, aid in its prevention.

## Materials and methods

### Study design and sample

This is a cross-sectional observational study on 74 adults outpatients with a diagnosis of ADHD. The patients were consecutively enrolled at the regional reference center for ADHD in the Psychiatry Unit of San Luigi Gonzaga University Hospital, Orbassano (Turin) *from September 2023 to March 2024*.

Aims and procedures were explained to all the enrolled patients. Inclusion criteria were: ≥ 18 years of age, diagnosis of ADHD according to DSM-5-TR criteria [31], written consent before participation Exclusion criteria were underage and refusal to participation. The study protocol was approved by the local Ethical Committees with number 939.140 The study was conducted in accordance with the Helsinki Declaration, as amended by the 64th WMA General Assembly in Fortaleza, Brazil, in October 2013.

### Assessment

Patients were assessed through a semi-structured interview administered upon the patient’s arrival at our clinic, during four outpatient visits (ranging from 3 to 5, depending on the complexity of the clinical picture and the availability of both the patients and their caregivers). Patients were clinically assessed by a trained psychiatrist, with the help of specific and validated tests. In detail, the semi-structured interview explored the following areas:

1) Sociodemographic data: age, sex, marital status, occupational status and education level;

2) Clinical features of ADHD: ADHD subtype; severity of symptoms in childhood and in adulthood (according to the “Diagnostic Interview for ADHD in adults” - DIVA, administered in the presence of a caregiver) [32]; current occurrence of symptoms (measured through ADHD rating scale IV – ADHD-RS IV) [33]; impulsiveness (measured through “Barratt Impulsiveness Scale” – BIS-11) [34]; ADHD related symptoms (such as mood swings, anger outbursts, low self-esteem, low tolerance for frustrations, sleep onset insomnia); areas of functional impairment (according to DIVA Criterion C); age at ADHD diagnosis; age at first ADHD treatment; family history of psychiatric disorders; physical activity, intensity of physical activity (≤ 3 h per week, 3–5 h per week, and ≥ 5 h per week).

3) Psychiatric comorbidities: the Italian version of the Structured Clinical Interview for DSM-5 Axis I Disorders (SCID-5) [35] was administered to assess psychiatric comorbidities, while personality status was evaluated clinically and using the Millon Clinical Multiaxial Inventory (MCMI-III) [36]. DSM-IV-TR Diagnoses were updated to meet the DSM-5-TR criteria [31].

4) Suicidality: the Italian version of the Columbia-Suicide Severity Rating Scale (C-SSRS) Lifetime/Recent version [7] was administrated to all the patients who met the criteria for ADHD diagnosis. C-SSRS consists of four subscales which explore SI severity, SI intensity, SB and lethality. SI severity ranges from 1 (wish to be dead) to 5 (active suicidal ideation with specific plan and intent). The SI intensity subscale explores frequency, duration, controllability, deterrents and reasons for SI. The third subscale includes SB (actual, interrupted and aborted suicide attempts, preparatory behaviors for a suicide attempt) and NSSIB. For our purpose, as other studies previously did [37, 38], lifetime SI was considered a score ≥ 1 on the severity subscale, while lifetime SSI was considered a severity score ≥ 4. This dichotomization relies on the assumption that the intent to act could be a predictive factor for future SB [7], allowing the identification of high-risk patients. Lifetime SB was considered a score ≥ 1 on the behavior subscale, as other studies previously did [7, 37]. This dichotomy was based on the fact that engaging in suicidal acts is associated with an increased risk of subsequent suicide attempts [39]. NSSIB was examined with a specific item on the behavior subscale.

### Statistical analysis

The sociodemographic and clinical features of the participants were summarized as mean and SD for continuous variables and as frequency and percentage for categorical variables. We tested the distribution of continuous variables using the Kolmogorov-Smirnov test.

The sample was divided, one at a time, in the following subgroups:

- ADHD with SI (score ≥ 1 on the suicidal severity subscale) vs. ADHD without SI (score = 0);
- ADHD with SSI (score ≥ 4 on the suicidal severity subscale) vs. ADHD without SSI (score < 4);
- ADHD with SB (preparatory acts or aborted/interrupted/actual attempts) vs. ADHD without SB;
- ADHD with NSSIB vs. ADHD without NSSIB.

Considering that the distribution was not normal (*P* < 0.001), comparisons were performed using χ^2^ tests for categorical variables and Kruskal-Wallis H test for continuous variables.

Binary logistic regression was used to identify explanatory variables related with lifetime SI/SSI/SB/NSSIB, considering the presence of lifetime SI/SSI/SB/NSSIB as the dependent variable. Significant variables were selected using a forward stepwise procedure. To be included in the equation, a probability of 0.05 was required. The group comparison results were presented as two-sided *p*-values rounded to three decimal places. The criterion for statistical significance in all comparison was a *p* value < 0.05.

All statistical analyses were performed by SPSS software version 29.0.1.0, *IBM Inc.*,* Armonk*,* New York.*

## Results

A total of 74 adult patients with a diagnosis of ADHD were enrolled in the study. The sample’s demographic and clinical features are shown in Table 1.

**Table 1:** Sociodemographic and clinical characteristics of the total sample (*n* = 74)

Sex, *n* (%)	
Male Female	50 (67.6) 24 (32.4)
Age, mean (SD)	30.05 (10.8)
Marital status, n (%)	
Single Married Separated Widowed	61 (82.4) 11 (14.9) 2 (2.7) 0 (0)
Education (years), mean (SD)	12.8 (3.4)
Paid employment, n (%)	
Yes No	56 (75.7) 18 (24.3)
Family history of psychiatric disorders, n (%)	30 (41.1)
Family history of ADHD, n (%)	
Yes No	9 (12.2) 65 (87.8)
Adult ADHD subtype, n (%)	
Inattentive subtype Combined subtype	32 (43.2) 42 (56.8)
Age at diagnosis (years), mean (SD)	25.9 (11)
Age at first ADHD treatment (years), mean (SD)	26.1 (10.3)
Ongoing ADHD treatment at assessment, n (%)	22 (29.7)
DIVA 2.0, mean (SD)	
Inattentive symptoms in childhood Hyperactivity symptoms in childhood Inattentive symptoms in adulthood Hyperactivity symptoms in adulthood	7.4 (1.1) 5.2 (2.9) 7.4 (1.2) 5.54 (2.5)
ADHD-RS, mean (SD)	36 (8.6)
BIS-11, mean (SD) Attentional impulsiveness Motor impulsiveness Non-planning impulsiveness	70.4 (11.6) 20.3 (4) 22.5 (4.7) 27.6 (5.2)
Lifetime psychiatric comorbidities, n (%)	
Any comorbid disorder Major depressive disorder Bipolar disorders Substance use disorders Personality disorders Anxiety disorders Autism spectrum disorders	51 (68.9) 27 (36.5) 3 (4.1) 19 (25.7) 8 (10.8) 6 (8.1) 5 (6.7)
Areas of functional impairment, n (%)	
Social functioning Relational functioning Academic functioning Occupational functioning	45 (60.8) 53 (71.6) 69 (93.2) 53 (71.6)
Related symptoms, n (%)	
Mood swings Anger outbursts Low self-esteem Low tolerance for frustrations Sleep onset insomnia	52 (70.3) 35 (47.3) 55 (74.3) 51 (68.9) 51 (68.9)

The lifetime prevalence of SI and SSI were 59.5% (*n* = 44) and 16.2% (*n* = 12), respectively. The 9.5% of patients (*n* = 7) showed lifetime SB, while NSSIB was found in 10.8% of the subjects (*n* = 8).

**Fig. 1:**
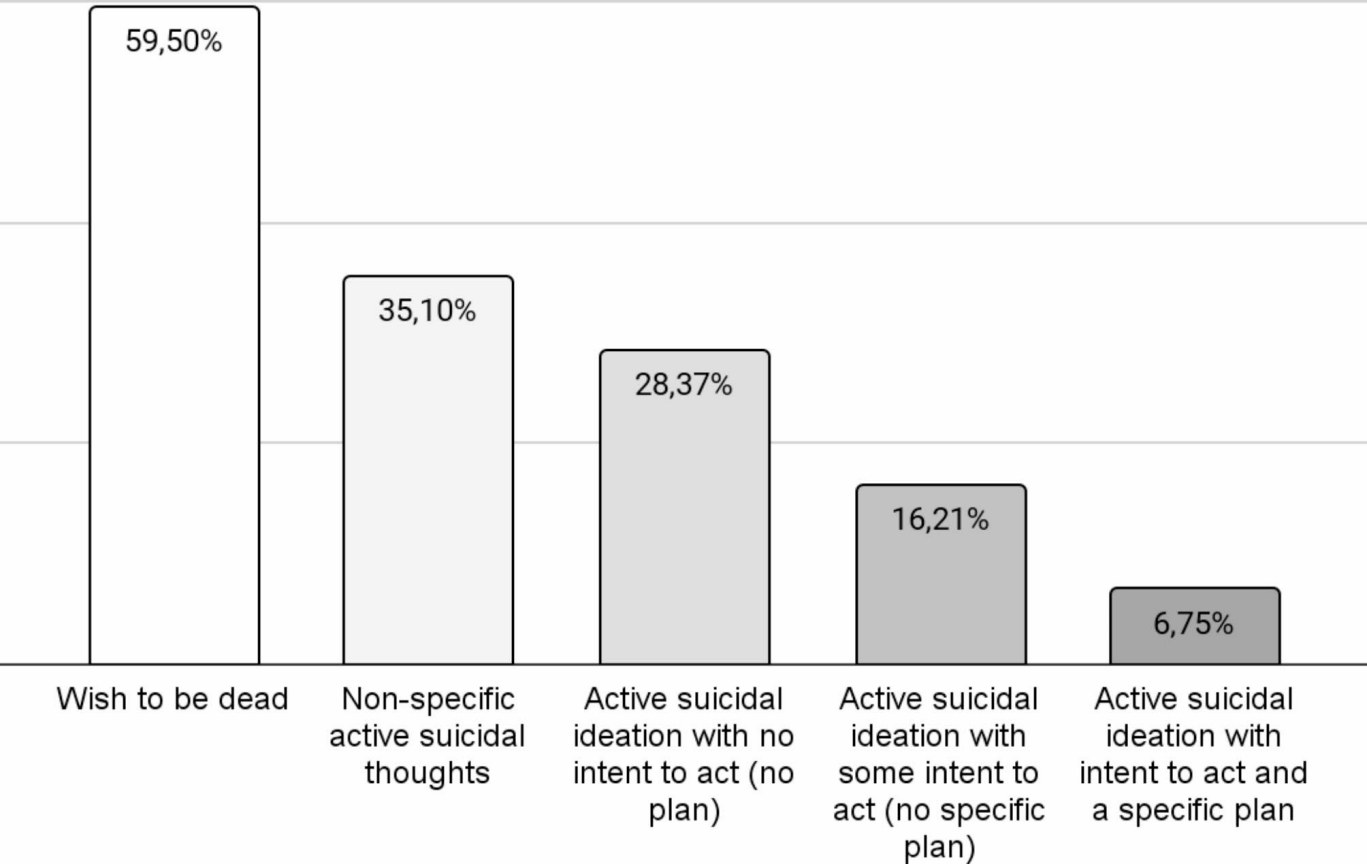
Cumulative distribution of the severity of suicidal ideation in individuals with ADHD (*n* = 74)

**Fig. 2:**
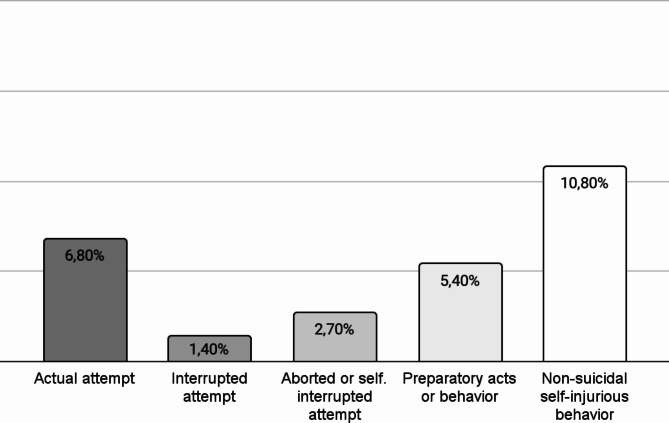
Different types of suicidal behaviors in individuals with ADHD (*n* = 74)

Figures 1 and 2 show the severity of lifetime SI and the different types of lifetime SB in the sample.

Tables 2, 3, 4 and 5 show the demographic and clinical features of the subgroups (ADHD with SI vs. ADHD without SI, ADHD with SSI vs. ADHD without SSI, ADHD with SB vs. ADHD without SB, ADHD with NSSIB vs. ADHD without NSSIB), compared according to χ^2^ tests or Kruskal-Wallis H test. The variables with a statistically significant difference were subjected to binary logistic regression.

**Table 2:** Comparison between ADHD with SI vs. ADHD without SI, according to χ2 tests for categorical variables and Kruskal-Wallis H test for continuous variables

	SI group (44)	NO SI group (30)	*p*-value
Sex, n (%)			
Male Female	27 (61.4) 17 (38.6)	23 (76.7) 7 (23.3)	0.167
Age (years), mean (SD)	27.66 (9.48)	30.03 (11.13)	0.328
Age at diagnosis (years), mean (SD)	25.02 (10.14)	27.27 (12.33)	0.395
Age at first ADHD treatment (years), mean (SD)	25.48 (9.1)	26.9 (12.06)	0.569
Ongoing ADHD treatment at assessment, n (%)	12 (27.3)	10 (33.3)	0.391
**Family history of psychiatric disorders**,** n (%)**	**23 (53.5)**	**7 (23.3)**	**0.010**
Family history of ADHD, n (%)	7 (15.9)	1 (3.3)	0.08
Adult ADHD subtype, n (%)			
Inattentive subtype Combined subtype	19 (43.2) 25 (56.8)	13 (43.3) 17 (56.7)	0.99
Childhood ADHD subtype, n (%)			
Inattentive subtype Combined subtype	15 (34.1) 29 (65.9)	11 (36.7) 19 (63.3)	0.82
DIVA 2.0, mean (SD)			
Inattentive symptoms in childhood Hyperactivity symptoms in childhood **Inattentive symptoms in adulthood** Hyperactivity symptoms in adulthood	7.55 (0.92) 5.16 (3.28) **7.84 (1.18)** 5.87 (2.64)	7.11 (1.41) 5.21 (2.53) **6.68 (1.1)** 5 (2.08)	0.185 0.956 **0.001** 0.228
ADHD-RS before treatment, mean (SD)	35.6 (7.8)	36.5 (9.72)	0.705
BIS-11, mean (SD) Attentional impulsiveness Motor impulsiveness Non-planning impulsiveness	71.27 (11.58) 20.8 (3.87) 23 (4.89) 27.41 (5,29)	69.27 (11.71) 19.53 (4.17) 21.73 (4.36) 28 (5.28)	0.469 0.187 0.258 0.638
Lifetime psychiatric comorbidities, n (%)			
Any comorbid disorder Major depressive disorder Bipolar disorders Substance use disorders Personality disorders Anxiety disorders Autism spectrum disorders	31 (70.4) 17 (38.6) 3 (6.8) 11 (25) 7 (15.9) 3 (6.8) 3 (6.8)	20 (66.7) 10 (33.3) 0 (0) 8 (26.7) 2 (6.6) 3 (10) 1 (3.3)	0.619 0.642 0.144 0.872 0.090 0.622 0.515
Areas of functional impairment, n (%)			
** Social functioning** Relational functioning Academic functioning Occupational functioning	**31 (70.5)** 34 (77.3) 42 (95.5) 32 (74.4)	**14 (46.6)** 19 (63.3) 27 (90) 21 (70)	**0.044** 0.192 0.359 0.721
Related symptoms, n (%)			
Mood swings Anger outbursts ** Low self-esteem** Low tolerance for frustrations Sleep onset insomnia	30 (68.2) 22 (50) **37 (84.1)** 30 (68.2) 29 (65.9)	22 (73.3) 13 (43.3) **18 (60)** 21 (70) 22 (73.3)	0.634 0.57 **0.02** 0.868 0.498

**Table 3:** Comparison between ADHD with SSI vs. ADHD without SSI, according to χ2 tests for categorical variables and Kruskal-Wallis H test for continuous variables

	SSI group (12)	NO SSI group (62)	*p*-value
Sex, n (%)			
Male Female	9 (18) 3 (12.5)	41 (82) 21 (87.5)	0.548
Age (years), mean (SD)	30.83 (11.82)	28.19 (9.88)	0.415
Age at diagnosis (years), mean (SD)	26.92 (10.33)	25.74 (11.26)	0.739
Age at first ADHD treatment (years), mean (SD)	28.67 (8.21)	25.52 (10.67)	0.339
Ongoing ADHD treatment at assessment, n (%)	2 (16.6)	20 (35.5)	0.129
**Physical activity**,** n (%)**	**3 (25)**	**35 (56.5)**	**0.046**
Family history of psychiatric disorders, n (%)	6 (50)	24 (39.3)	0.493
Family history of ADHD, n (%)	1 (12.5)	7 (11.5)	0.750
**Number of hospitalizations**,** mean (SD)**	**1.17 (1.64)**	**0.24 (0.93)**	**0.008**
Adult ADHD subtype, n (%)			
Inattentive subtype Combined subtype	4 (33.3) 8 (66.7)	28 (45.2) 34 (54.8)	0.449
Childhood ADHD subtype, n (%)			
Inattentive subtype Combined subtype	4 (33.3) 8 (66.7)	22 (35.5) 40 (64.5)	0.886
DIVA 2.0, mean (SD)			
**Inattentive symptoms in childhood** Hyperactivity symptoms in childhood Inattentive symptoms in adulthood Hyperactivity symptoms in adulthood	**8.10 (0.74)** 5.80 (3.22) 8 (0.94) 6.7 (2.16)	**7.2 (1.16)** 5.03 (2.95) 7.25 (1.31) 5.25 (2.46)	**0.024** 0.470 0.097 0.096
ADHD-RS before treatment, mean (SD)	35.9 (9.09)	36.04 (8.65)	0.963
**BIS-11**,** mean (SD)** ** Attentional impulsiveness** ** Motor impulsiveness** Non-planning impulsiveness	**79 (7.19)** **22.83 (4.01)** **25 (3.24)** 29.7 (3.9)	**68.81 (11.6)** **19.79 (3.86)** **22 (4.8)** 27.98 (5.24)	**0.005** **0.015** **0.042** 0.08
Lifetime psychiatric comorbidities, n (%)			
Any comorbid disorder Major depressive disorder Bipolar disorders Substance use disorders Personality disorders Anxiety disorders Autism spectrum disorders	11 (91.7) 5 (41.7) 1 (33.3) 4 (33.3) 2 (16.7) 0 (0) 1 (8.3)	40 (65.6) 22 (35.5) 2 (3.2) 15 (24.2) 6 (9.7) 6 (9.7) 3 (4.8)	0.072 0.684 0.412 0.507 0.475 0.261 0.624
Areas of functional impairment, n (%)			
Social functioning Relational functioning Academic functioning Occupational functioning	10 (83.3) 10 (83.3) 10 (83.3) 10 (83.3)	35 (56.5) 43 (69.4) 59 (95.2) 43 (70.5)	0.081 0.326 0.135 0.574
Related symptoms, n (%)			
Mood swings Anger outbursts Low self-esteem Low tolerance for frustrations Sleep onset insomnia	11 (91.7) 7 (58.3) 10 (83.3) 9 (75) 8 (66.7)	41 (78.8) 28 (45.2) 45 (72.6) 42 (67.7) 43 (69.4)	0.076 0.403 0.435 0.619 0.854

**Table 4:** Comparison between ADHD with SB vs. ADHD without SB, according to χ2 tests for categorical variables and Kruskal-Wallis H test for continuous variables

	SB group (7)	NO SB group (67)	*p*-value
Sex, n (%)			
Male Female	4 (57.1) 3 (42.9)	46 (68.7) 21 (31.3)	0.536
Employment, n (%)			
** Unemployed** ** Employed** ** Student** ** Retired**	**5 (71.4)** **1 (14.2)** **1 (14.2)** **0 (0)**	**13 (19.5)** **32 (47.7)** **21 (31.3)** **1 (1.5)**	**0.048**
Age (years), mean (SD)	33.57 (12.55)	28.10 (9.86)	0.178
Age at diagnosis (years), mean (SD)	26.86 (10.76)	25.84 (11.16)	0.818
Age at first ADHD treatment (years), mean (SD)	27 (7.91)	25.94 (10.59)	0.798
Ongoing ADHD treatment at assessment, n (%)	2 (28.6)	21 (31.3)	0.880
Family history of psychiatric disorders, n (%)	4 (57.1)	26 (39.4)	0.364
Family history of ADHD, n (%)	0 (0)	8 (12,1)	0.329
**Number of hospitalizations**,** mean (SD)**	**1.86 (1.86)**	**0.24 (0.9)**	**< 0.001**
Adult ADHD subtype, n (%)			
Inattentive subtype Combined subtype	2 (28.5) 5 (71.5)	31 (46.3) 36 (53.7)	0.104
Childhood ADHD subtype, n (%)			
Inattentive subtype Combined subtype	2 (28.5) 5 (71.5)	25 (37.3) 42 (62.7)	0.225
DIVA 2.0, mean (SD)			
Inattentive symptoms in childhood Hyperactivity symptoms in childhood Inattentive symptoms in adulthood Hyperactivity symptoms in adulthood	8.2 (0.84) 6.5 (2.34) 8.1 (1.09) 6.9 (1.92)	7.29 (1.14) 4.98 (3) 7.31 (1.27) 5.36 (2.46)	0.090 0.154 0.142 0.112
ADHD-RS before treatment, mean (SD)	37.2 (8.34)	35.9 (8.74)	0.752
**BIS-11**,** mean (SD)** Attentional impulsiveness ** Motor impulsiveness** Non-planning impulsiveness	**79.3 (7.95)** 22.71 (4.3) **25.57 (2.82)** 30,57 (5,25)	**69.54 (11.57)** 20 (3.93) **22.16 (4.75)** 27,34 (5,2)	**0.033** 0.093 **0.050** 0,123
Lifetime psychiatric comorbidities, n (%)			
Any comorbid disorder Major depressive disorder Bipolar disorders Substance use disorders Personality disorders Anxiety disorders Autism spectrum disorders	7 (100) 3 (42.9) 1 (14.3) 3 (42.9) 2 (28.6) 0 (0) 1 (14.3)	44 (66.7) 24 (35.8) 3 (4.5) 16 (23.9) 6 (9) 6 (9) 3 (4.5)	0.068 0.713 0.345 0.274 0.112 0.409 0.275
Areas of functional impairment, n (%)			
Social functioning Relational functioning Academic functioning Occupational functioning	6 (85.7) 6 (85.7) 6 (85.7) 6 (85.7)	39 (58.2) 47 (70.1) 63 (94) 46 (69.7)	0.156 0.385 0.232 0.404
Related symptoms, n (%)			
Mood swings Anger outbursts Low self-esteem Low tolerance for frustrations Sleep onset insomnia	6 (85.7) 4 (57.1) 5 (71.4) 6 (85.7) 5 (71.4)	46 (68.7) 30 (44.8) 50 (74.6) 45 (67.2) 46 (68.7)	0.347 0.179 0.854 0.313 0.880

**Table 5:** Comparison between ADHD with NSSIB vs. ADHD without NSSIB, according to χ2 tests for categorical variables and Kruskal-Wallis H test for continuous variables

	NSSIB group (8)	NO NSSIB group (66)	*p*-value
Sex, n (%)			
Male Female	3 (37.5) 5 (62.5)	47 (71.2) 19 (28.8)	0.06
Age (years), mean (SD)	29.5 (11.52)	30.12 (10.83)	0.79
Age at diagnosis (years), mean (SD)	25.88 (12.84)	25.94 (10.93)	0.988
Age at first ADHD treatment (years), mean (SD)	25.88 (12.84)	26.06 (10.09)	0.962
Ongoing ADHD treatment at assessment, n (%)	2 (25)	21 (31.8)	0,888
**Current smoking**,** n (%)**	**5 (62.5)**	**17 (25.8)**	**0.032**
Family history of psychiatric disorders, n (%)	3 (37.5)	27 (41.5)	0.827
Family history of ADHD, n (%)	1 (12.5)	7 (10.6)	0,871
**Number of hospitalizations**,** mean (SD)**	**1.38 (1.99)**	**0.27 (0.92)**	**0.008**
Adult ADHD subtype, n (%)			
Inattentive subtype Combined subtype	2 (25) 6 (75)	30 (45.5) 36 (54.5)	0,270
Childhood ADHD subtype, n (%)			
Inattentive subtype Combined subtype	2 (25) 6 (75)	24 (36.4) 42 (63.6)	0,525
DIVA 2.0, mean (SD)			
Inattentive symptoms in childhood Hyperactivity symptoms in childhood Inattentive symptoms in adulthood Hyperactivity symptoms in adulthood	8.17 (0.75) 6.33 (3.14) 8.17 (0.98) 6.67 (3.32)	7.27 (1.14) 5.02 (2.97) 7.3 (1.28) 5.39 (2.32)	0.070 0.320 0.118 0.235
ADHD-RS before treatment, mean (SD)	37 (9.67)	35.92 (8.64)	0.793
BIS-11, mean (SD) Attentional impulsiveness Motor impulsiveness Non-planning impulsiveness	74.5 (11.85) 21.13 (4.45) 23.25 (4.89) 30.13 (4.99)	69.97 (11.56) 20.18 (3.99) 22.39 (4.7) 27.3 (5.24)	0.300 0.535 0.630 0.160
Lifetime psychiatric comorbidities, n (%)			
Any comorbid disorder Major depressive disorder Bipolar disorders Substance use disorders ** Personality disorders** Anxiety disorders Autism spectrum disorders	4 (50) 2 (25) 0 (0) 1 (12.5) **3 (37.5)** 0 (0) 0 (0)	48 (72.7) 25 (37.9) 3 (4.5) 10 (15.2) **5 (7.6)** 6 (9.1) 4 (6.1)	0.184 0.475 0.538 0.842 **0.010** 0.374 0.474
Areas of functional impairment, n (%)			
Social functioning Relational functioning Academic functioning Occupational functioning	5 (62.5) 6 (75) 7 (87.5) 6 (75)	40 (60.6) 47 (71.2) 62 (93.9) 47 (72.3)	0.917 0,822 0.493 0.493
Related symptoms, n (%)			
Mood swings Anger outbursts Low self-esteem Low tolerance for frustrations Sleep onset insomnia	6 (75) 6 (75) 5 (62.5) 5 (62.5) 5 (62.5)	46 (69.7) 29 (43.9) 50 (75.8) 46 (69.7) 46 (69.7)	0.757 0.097 0.418 0.678 0.678

The results of the binary logistic regression models are described in Tables 6, 7 and 8, and 9.

**Table 6:** The relationship between potential explanatory variables and lifetime suicidal ideation: results from the binary logistic regression analysis (*n* = 74)

	B	SE	Wald	*p*-value	OR	95%CI
**Severity of inattentive symptoms in adulthood**	**0.829**	**0.360**	**5.317**	**0.021**	**2.291**	**0.164–2.884**
Family history of psychiatric disorders	1.029	0.588	3.068	0.080	2.800	-0.161–2.680
**Impairment in social functioning** **Low self-esteem**	**1.075** **1.374**	**0.548** **0.614**	**3.856** **5.013**	**0.049** **0.025**	**2.931** **3.953**	**0.037–2.672** **0.104–3.283**
Constant	2.405	0.680	12.512	< 0.001	11.080	-

**Table 7:** The relationship between potential explanatory variables and lifetime severe suicidal ideation: results from the binary logistic regression analysis (*n* = 74)

	B	SE	Wald	*p*-value	OR	95%CI
**Severity of inattentive symptoms in childhood**	**1.772**	**0.815**	**4.725**	**0.030**	**5.880**	**0.291–65.893**
Total impulsivity **Attentional impulsiveness** Motor impulsiveness	0.185 **0.845** -0.311	0.193 **0.406** 0.330	0.920 **4.327** 0.889	0.337 **0.038** 0.346	1.203 **20.346**0.327	-5.430–70.744 **0.039–2.176** -102.161–3.571
**Number of hospitalizations**	**0.587**	**0.261**	**5.050**	**0.025**	**1.799**	**0.030-38.569**
**Physical activity**	**-2.641**	**1.069**	**6.104**	**0.013**	**0.071**	**-80.768- -0.594**
Constant	-14.133	4.646	9.254	0.002	0.000	-

**Table 8:** The relationship between potential explanatory variables and lifetime suicidal behavior: results from the binary logistic regression analysis (*n* = 74)

	B	SE	Wald	*p*-value	OR	95%CI
Employment	-0.398	0.374	1.132	0.287	0.672	-25.777–0.876
Total impulsivity Motor impulsiveness	0.118 -0.090	0.096 0.213	1.503 0.178	0.220 0.673	1.125 0.914	-0.316–0.862 − 1.431–2.980
Number of hospitalizations	0.593	0.265	5.001	0.080	1.810	-0.013–49.540
Constant	-8.845	4.222	4.389	0.036	0.000	-

**Table 9:** The relationship between potential explanatory variables and lifetime non suicidal self-injury behavior: results from the binary logistic regression analysis (*n* = 74)

	B	SE	Wald	*p*-value	OR	95%CI
Current smoking	1.043	0.909	1.316	0.251	2.838	-2.066–19.891
Personality disorders	1.640	0.940	3.048	0.081	5.157	-18.844–20.923
Number of hospitalizations	0.299	0.274	1.186	0.276	1.348	-17.326–1.454
Constant	-3.076	0.646	22.700	< 0.001	0.046	-

The incidence of lifetime SI appeared related to severity of inattentive symptoms during adulthood (*p* = 0.021, OR 2,0.291, 95%CI 0.164–2.884), low self-esteem (*p* = 0.025, OR 3.953, 95%CI 0.104–3.283) and impairment in social functioning (*p* = 0.049, OR 2.931, 95%CI 0.037–2.672).

Lifetime SSI was significantly associated to the severity of inattentive symptoms during childhood (*p* = 0.030, OR 5.880, 95%CI 0.291–65.893), attentional impulsiveness (*p* = 0.038, OR 2.327, 95%CI 0.039–2.176) and number of hospitalizations (*p* = 0.025, OR 1,799, 95%CI 0.030-38.569). Physical exercise showed to be related with a significant lower lifetime prevalence of SSI (*p* = 0.013, OR 0.071, 95%CI − 80.768- -0.594).

No socio-demographic features resulted significantly associated to the occurrence of SI and SSI.

The prevalence of lifetime SB did not appear significantly related to any socio-demographic or clinical feature (including any psychiatric comorbidity), also when stratified in the different types of SB. Similarly, no factor significantly linked with higher prevalence of NSSIB emerged.

## Discussion

This observational study aimed to evaluate the prevalence of suicidality (SI, SSI, SB, and NSSIB) in adult patients with ADHD using a dimensional approach and a validated instrument; we also analyzed socio-demographic and clinical factors potentially related to occurrence of SI, SSI, SB, or NSSIB in these patients.

The Columbia-Suicide Severity Rating Scale (C-SSRS) has enabled us to more precisely define and quantify the complex phenomenon of suicidality, which cannot be captured by a single question. As emphasized by Posner and colleagues in the original validation findings of this scale, a general desire to be dead does not pose a comparable risk factor for SB when contrasted with active SI [7]. Additionally, given the high levels of impulsivity in patients with ADHD and the associated risk of acting out, identifying those with active SI could be crucial in preventing suicide.

We identified a high prevalence of suicidality in adult patients with ADHD. Specifically, 59.5% of our sample reported wishing to be dead at least once in their life; moreover, 9.5% of the participants reported at least one lifetime SB. A considerable proportion of individuals with ADHD can be considered at high risk for suicide: 16.2% of our sample scored ≥ 4 on the severity scale of the C-SSRS, presenting lifetime active SI with a specific plan and intent (6.8%) or active SI with some intent to act but no plan (9.4%). Concerning SB, only a minority of our patients had actually attempted suicide (6.8%); many others, however, engaged in some SB, such as interrupted attempts (1.4%), aborted or self-interrupted attempts (2.7%), or in preparatory acts or behaviors (5.4%). Moreover, a considerable part of our sample (10.8%) engaged in NSSIB. Our results are reasonably in line with findings from a recent meta-analysis showing the lifetime prevalence of SI and SB to be 40% and 18.9%, respectively [6].

The identification of a high suicidality risk among adults with ADHD underscores the necessity for focused assessment and careful monitoring within clinical practice. Regular clinical assessments and implementation of psychoeducational interventions, not only for patients but also for their familial and caregiving networks, can be crucial tools in addressing this complex clinical concern. It is essential to assess suicidality in patients with ADHD in a dimensional manner during the initial evaluation, employing both clinical judgment and, where feasible, specific tools such as the Columbia Suicide Severity Rating Scale. For patients exhibiting a heightened risk profile, suicidality should not only be monitored as an ongoing component but also integrated into clinical outcomes and management strategies. This includes considering the use of medications that may have transdiagnostic suicidal effects, such as lithium salts [40], particularly in cases where the risk is deemed elevated.

The severity of inattentive symptoms in adulthood appeared significantly associated with lifetime SI, while the severity of hyperactivity/impulsivity symptoms did not result to have an impact on suicidality (both SI, SSI, SB and NSSIB). This evidence suggests that the association between ADHD and suicidality could be mediated by inattention, which represents the core symptom of ADHD.

In accordance with this hypothesis, the only type of impulsiveness (measured through BIS-11) which resulted associated with suicidality (specifically with SSI) in our sample was attentional impulsiveness. This has been defined as an inability to focus attention or concentrate, and it assesses task-focus, intrusive thoughts, and racing thoughts [41]. Instead, motor impulsiveness (acting without thinking) and non-planning impulsiveness (lack of “futuring” or forethought) did not result to affect both SI and SB, confirming that the risk of suicide in ADHD patients could depend on inattention rather than impulsivity itself.

Furthermore, while lifetime SI appeared related to the severity of inattentive symptoms in adulthood, we found that lifetime SSI was associated with the severity of inattentive symptoms during childhood. This result endorses the potential impact in adulthood of the symptoms during childhood, *underlining that* the intensity of symptoms during this critical developmental period plays a more crucial role in influencing suicidality risk than the timing of the diagnosis itself. Previous research has identified several predictive factors for suicidality in ADHD, such as early externalizing behaviors, adverse childhood experiences, and negative father-daughter interactions, while not placing emphasis on inattentive symptoms [42]. Therefore, considering SSI as the proper risk factor for suicide, exploring symptoms of attention deficit in childhood should be a target in ADHD patients. However, in clinical practice, identifying this cluster in childhood can be challenging due to the occasional unavailability of caregivers and the less overt presentation of inattention, which is not always the predominant feature of ADHD, particularly in children.

It is important to underline that in our sample SI, SSI, SB and NSSIB did not appear associated with any psychiatric comorbidity, in line with prior research findings from Septier’s et al. meta-analysis [6].

Furthermore, we did not find any clinical or socio-demographic factors significantly associated with SB and NSSIB in adult patients with ADHD, indicating a direct correlation between these phenomena.

Unlike SB and NSSIB, other clinical factors were found to be associated with SI and SSI in addition to the previously mentioned inattentive symptoms and attentional impulsiveness. Patients with SI exhibited significantly more frequent impairments in social functioning, though not in other areas. This finding, consistent with existing literature, supports the notion of a causal link between social isolation and suicide, as well as the protective influence of social support against suicide [43]. Physical activity appeared to be associated with a lower lifetime prevalence of SSI, suggesting that being physically active could reduce suicidal risk. While engaging in physical activity has proven to be a protective factor against SSI, the intensity of the activity did not appear to be correlated with the risk, suggesting that even light-intensity physical activity can be protective. This evidence aligns with a recent meta-analysis conducted on psychiatric patients [44] and is significant considering that there are only a limited number of interventions that have demonstrated effectiveness against suicide, and these are not always accessible within public health systems. Indeed, only a few medications have been shown to clearly reduce suicidal risk, such as lithium, ketamine and clozapine [45, 46]. Meanwhile, interventions like cognitive-behavioral therapy have proven effective but are often not readily available within public health systems [45].

Low self-esteem is a known risk factor for suicide, especially in emerging adulthood [47]. It appeared significantly related with lifetime SI (it was found in 84.1% of the patients with suicidality history), but it is important to underline how frequently it occurs also in patients without an history of SI/SSI/SB/NSSIB (60%), being one of the most associated symptoms in ADHD.

No gender differences emerged regarding both SI, SSI, SB and NSSIB in our sample.

Our study has several strengths, including a well-characterized clinical sample comprised of carefully diagnosed patients with thorough evaluations of their ADHD symptoms and comorbidities, the use of standardized validated assessments, and a dimensional approach. However, our study should be considered in light of some limitations. First, the cross-sectional design does not allow for the inference of causal relationships or the evaluation of etiological factors. Another limitation of our study is the gender ratio of approximately 2:1 (males to females) among the patients, which, although slightly higher than the 1.7:1 ratio reported in the literature [48], is still consistent with the general epidemiology of the disorder. Furthermore, while we addressed various aspects of emotional dysregulation (such as anger control, mood reactivity, and frustration tolerance), we did not comprehensively cover emotional dysregulation in its entirety. The absence of patients with the predominantly hyperactive-impulsive subtype and the high prevalence of single patients represent a potential additional barrier to the generalization of our results, as well as the low prevalence of familial ADHD in our sample limits our ability to generalize the results and precludes us from excluding a potential protective role of familial ADHD in suicidality. Additionally, the sample did not include individuals who had completed suicide, which means we cannot determine if the results apply to suicide deaths. Another significant limitation of this study is the small number of participants, which renders the results regarding potential predictors of SB/NSSIB preliminary. Additionally, the absence of a comparison group from the general population further limits the ability to contextualize these findings. It is, therefore, not possible to rule out the possibility that the risk of SB and NSSIB is mediated by factors such as personality disorders or specific temperaments. These traits are particularly prevalent among adults with ADHD [49], contributing to greater functional impairment and reduced treatment response [50], and have also been associated with suicidality dimensions [8].

Despite these limitations, our findings are noteworthy since they highlight that a significant proportion of patients with ADHD have lifetime SI/SB. Moreover, this association appeared not to be affected by psychiatric comorbidities. Instead, our findings suggest that the risk of suicide in ADHD patients could depend on inattention itself. Interestingly, this dimension may be more closely associated with suicidality severity due to several interrelated psychological and social factors. Specifically, individuals exhibiting higher levels of inattention might experience lower self-esteem, a pervasive sense of hopelessness, limited opportunities for personal and professional development, decreased engagement in social situations, and heightened feelings of isolation. These elements can create a detrimental emotional environment that exacerbates suicidality risk, suggesting a complex interplay between ADHD symptoms and the psychosocial challenges faced by these individuals. In conclusion, adult patients with ADHD can be considered at risk of suicide, and it is important to identify which patients are at higher risk, in order to guide preventive pharmacological or psychological treatments and psychoeducational interventions. Therefore, for adult ADHD patients, alongside pharmacological therapy, the utilization of psychotherapeutic interventions, particularly cognitive-behavioral and psychoeducational approaches, is crucial. These interventions aid patients in gaining a deeper understanding of their condition, enhancing self-esteem, and guiding them towards adopting healthy and protective lifestyles, such as regular physical activity.

Future research should aim to replicate our findings in a larger sample to enhance generalizability and robustness. Additionally, it would be beneficial to integrate cognitive assessments specifically focused on the attentional dimension, as well as other cognitive measures, to further explore their associations with dimensions of suicidality. Furthermore, it would be valuable to examine how previous psychotherapy experiences, as well medications and psychosocial interventions (whether individual or family-based) influence suicidality. Such investigations could provide important insights into effective management strategies for individuals with ADHD.

## Data Availability

No datasets were generated or analysed during the current study.
